# The Descriptive and Disproportionality Assessment of EudraVigilance Database Reports on Capecitabine Induced Cardiotoxicity

**DOI:** 10.3390/cancers16223847

**Published:** 2024-11-16

**Authors:** Razvan Constantin Vonica, Anca Butuca, Andreea Loredana Vonica-Tincu, Claudiu Morgovan, Manuela Pumnea, Remus Calin Cipaian, Razvan Ovidiu Curca, Florina Batar, Vlad Vornicu, Adelaida Solomon, Adina Frum, Carmen Maximiliana Dobrea, Dan Damian Axente, Felicia Gabriela Gligor

**Affiliations:** 1 Preclinical Department, Faculty of Medicine, “Lucian Blaga” University of Sibiu, 550169 Sibiu, Romania; razvanconstantin.vonica@ulbsibiu.ro (R.C.V.); claudiu.morgovan@ulbsibiu.ro (C.M.); manuelapumnea@yahoo.com (M.P.); florina.batar@ulbsibiu.ro (F.B.); adina.frum@ulbsibiu.ro (A.F.); carmen.dobrea@ulbsibiu.ro (C.M.D.); felicia.gligor@ulbsibiu.ro (F.G.G.); 2 Clinical Department, Faculty of Medicine, “Lucian Blaga” University of Sibiu, 550169 Sibiu, Romania; calin.cipaian@ulbs.ro (R.C.C.); solomonadelaida@gmail.com (A.S.); 3 County Clinical Emergency Hospital of Sibiu, 2-4 Corneliu Coposu Str., 550245 Sibiu, Romania; 4 Department of Oncology, “Elysee Hospital”, 510040 Alba Iulia, Romania; razvancurca@gmail.com; 5 Department IX Surgery, Discipline of Oncology, Faculty of Medicine, Victor Babes University of Medicine and Pharmacy Timisoara, Eftimie Murgu Square 2, 300041 Timisoara, Romania; vornicuvlad91@gmail.com; 6 Fifth Surgical Clinic, “Iuliu Hatieganu” University of Medicine and Pharmacy, 400012 Cluj-Napoca, Romania; damian.axente@umfcluj.ro

**Keywords:** colon cancer, cardiotoxicity, fluoropyrimidines, capecitabine, adverse drug reactions, pharmacovigilance, EudraVigilance

## Abstract

Capecitabine (CAP) is one of the most commonly prescribed fluoropyrimidines in oncology, especially in the treatment of colon cancer. Cardiac toxicity is a severe and potentially lethal adverse drug reaction (ADR) against fluoropyrimidines. Cardiac ADRs, such as myocardial infarction (MI), heart failure (HF), arrhythmias, and a number of cardiomyopathies, are reported for these molecules. To have a better understanding of the risk–benefit ratio of colon cancer therapy, a pharmacovigilance study of real-world evidence of the cardiac toxicity of antineoplastic agents is required. Aim: This post-marketing research on CAP aims to assess the risk of cardiac toxicity. Five other antitumor drugs used in colorectal cancer, i.e., 5-fluorouracil (5-FU), irinotecan (IRI), oxaliplatin (OX), bevacizumab (BEV) and panitumumab (PAN), were also studied to create a relative profile of observed cardiotoxicity. Methods: A retrospective study based on reports submitted in the EudraVigilance (EV) database until 28 July 2024 was conducted. Using the aggregated data from EV, a descriptive analysis and disproportionality analysis of cardiac ADRs induced by fluoropyrimidines were performed. To evaluate the disproportionality of the signals, Reporting Odds Ratio (ROR) and 95% confidence interval (95% CI) were calculated by comparison with other drugs used in colorectal cancer: 5-FU, IRI, OX, BEV, and PAN. Results: “Cardiac disorders” represent 3.4% of the total reports for CAP. The value is comparable to 5-FU, but higher than for other drugs. t was observed that there are no significant differences in the occurrence of cardiac ADRs in patients exposed to CAP and 5-FU treatments, and in particular MI and HF. Compared to 5-FU, which could produce cardiac arrythmias with a higher probability than all other drugs, CAP has a higher probability of reporting this ADR only in comparison with IRI (ROR: 1.2971; 95% CI: 1.0196-1.6502). Conclusions: CAP induces adverse cardiovascular reactions, especially MI, HF, and cardiomyopathies. Arrhythmias have been shown to be side effects more frequent associated with 5-FU than with CAP. The results emphasize the need for a rigorous cardiovascular monitoring of patients following treatment with CAP or 5-FU and especially for those with pre-existing cardiac pathology.

## Simple Summary

Capecitabine (CAP), belonging to the fluoropyrimidines class, is one of the most common drugs used in the treatment of colon cancer. In this study we cover the real-world impact of adverse effects, with focus on cardiotoxicity. The frequency of reports of cardiac toxicity in the EudraVigilance database was studied. Following the analysis, we observed that CAP and 5-FU can cause heart diseases, such as acute myocardial infarction, angina pectoris, heart failure, etc. From a physio-pathological point of view, coronary vasospasm, endothelial dysfunction and oxidative stress are the main factors in the production of cardiotoxicity induced by fluoropyrimidines.

## 1. Introduction

Colon cancer is situated in the first half of the top 10 most common cancer forms. Among the newly diagnosed cancers, the frequency of colon cancer is 12%, according to the International Agency for Cancer Research. 40,000 cases are diagnosed in the United Kingdom each year [1]. It is known that the etiology is multifactorial, including (i) environmental factors, such as high intake of red meat, high consumption of fats and processed meat, low consumption of fruits and vegetables and fibers, (ii) pathological conditions: obesity, inflammatory bowel diseases, diabetes, etc. [2], (iii) genetic factors: familial adenomatous polyposis (FAP), hereditary non-polyposis colorectal cancer (NHPCC), etc. [3]. The information found in the Global Cancer Observatory (GLOBOCAN) demonstrates a significant increase in cancer cases, with 19.98 million new cases registered in 2022 and 9.74 million patients who died of this disease [4]. The prevalence of survivors after diagnosis at 5 years with colorectal cancer was 50.6 million [1].

Colorectal cancer can be prevented through large-scale screening programs. This approach has had a good impact, triggering a decrease in the illness in countries with developed medical systems [5]. Historically, until the mid-1990s, colon cancer patients were treated with 5-Fluorouracil (5-FU) [6]. Along with the progress of medical technology, the pharmacotherapy for colon cancer has become more and more complex and effective, as there are drugs available from a variety of classes, which include (i) cytotoxic agents (oxaliplatin—OX and irinotecan—IRI) [7]; (ii) oral fluoropyrimidines, which bring benefits to the patients’ quality of life due to the fact that the treatment can be administered at home (capecitabine—CAP); (iii) biological agents (bevacizumab—BEV, panitumumab—PAN, pembrolizumab, nivolumab) etc. [8]. More recently, anti-angiogenic agents, such as Ziv (aflibercept and regorafenib) were approved and introduced [9].

The most frequently used drugs in the treatment of colon cancer are fluoropyrimidines, such as 5-FU and CAP. These drugs have proven their effectiveness if they are also used in combination with other drugs, such as (i) FUFOL (5-FU + Leucovorin (LV)), (ii) CAPOX (CAP+ OX), (iii) FOLFOX (5-FU + OX + LV), (iv) FOLFIRI (5-FU + IRI + LV) or (v) FOLFOXIRI (5-FU + OX + IRI + LV) [1].

Following the use of fluoropyrimidines on a large scale, a series of side effects have been observed, among which the most severe is cardiac toxicity [10,11]. Coronary vasospasm is the most frequent and severe cardiac toxicity that can occur after treatment with 5-FU or CAP [12]. This manifestation can cause different degrees of cardiac ischemia, accompanied by angina pectoris, myocardial infarction or even sudden death [10]. Angina pectoris can be typical or atypical and represents the most frequent manifestation of cardiac toxicity post-administration of CAP [13,14].

In general, the cardiotoxicity of exposure to fluoropyrimidines occurs during the first administration cycle [15,16,17]. The first symptoms appear most frequently 12 h after starting the infusion with 5-FU, or in the first 2–3 days after taking, but cardiotoxicity can appear at any time, even 1–2 days after the infusion or longer [18]. Astrup et al. studied 106 patients to whom 5-FU was administered by infusion in the short-term in the FOLFOX regimen, and nine of the studied patients had angina pectoris during the treatment [19]. Acute symptoms can start with angina pectoris, cardiac arrhythmias, and hypertension. From the point of view of chronic cardiotoxicity, this can become permanent and cumulative, being represented by heart failure [20].

Drug studies based on pharmacovigilance bring important benefits in clinical practice with the aim of personalizing and minimizing possible adverse reactions. In this way, health professionals will promptly recognize and treat possible adverse events by applying prophylactic, diagnostic or therapeutic measures to patients exposure to these types of injuries. Cardiac toxicity monitoring is essential in making early intervention more efficient, with the aim of reducing the mortality of patients treated with fluoropyrimidines [21]. The development of new clinical guidelines and monitoring protocols are supported by pharmacovigilance studies, which contribute to improving the quality and lifespan of patients [22]. 

To expand the knowledge of cardiotoxicity risk factors during fluoropyrimidine treatment, this research aimed to assess spontaneously reported cardiac adverse reactions following the use of CAP by investigating the EudraVigilance (EV) database. The safety profile of CAP comprises the identification and evaluation of adverse effects [23].

## 2. Materials and Methods

### 2.1. Study Design

A descriptive and disproportionality analysis of spontaneous ADRs reported for CAP were performed. For comparison, other antitumor drugs used in colorectal cancer were chosen. The analysis included all reports registered on the portal adrreports.eu, starting with the first report for each drug: CAP (28 January 2003), 5-FU (4 February 2003), IRI (11 February 2003), OX (26 August 2003), BEV (16 April 2004), and PAN (20 November 2006), until 28 July 2024 [24]. Data included in the Individual Case Safety Reports (ICSRs) were extracted between 1 and 3 August 2024. Health Professionals (HP) or Non-HP can fill ICSRs for patients originating from the European Economic Area (EEA) or Non-EEA [25].

### 2.2. Material

According to EMA regulations, different preferred terms (PTs), including 27 System Organ Classes (SOCs), can be used for reporting the ADRs in ICSRs. The Medical Dictionary for Regulatory Activities (MedDRA) is a hierarchic structure of different categories of terms classified as “medical and health-related”. Thus, PTs represent a medical terminology used for the coding of the ADRs. They are preferred when presenting the unique adverse effects or clinical conditions reported in databases. On the other hand, SOC is the highest level of hierarchy and includes terms grouped according to the organ system affected [26].

Cardiotoxicity of CAP includes arrhythmias, heart failure (HF), cardiomyopathy, and myocardial infarction (MI). Thus, to evaluate cardiotoxicity, some PTs related to different medical conditions have been selected (Table 1).

Data were extracted from ICSRs containing at least one PT from Table 1, used for reporting cardiotoxicity.

### 2.3. Data Analysis

#### 2.3.1. Descriptive Analysis

General characteristics included in ICSRs (patients’ age, sex, geographical origin, reporter) were analyzed [27]. Subsequently, a comparative analysis of reports related to CAP use with other antitumoral drugs for colorectal cancers (5-FU, BEV, IRI, OX, and PAN) was performed. Thus, (i) the ratio of ADRs reported for each ICSR, (ii) the structure of ADRs by seriousness, (iii) the distribution of ADRs by SOCs, and (iv) the distribution of ADRs by outcome were compared. An ADR is classified as serious when it is life-threatening, and/or it determines significant incapacity, and/or causes congenital anomalies [28]. Some outcomes indicate a progression of patient status (R—recovered, RS—resolved, RG—recovering, RSG—resolving), while others reflect an unfavorable progression (NR—not recovered, NRS—not resolved, or recovered/resolved with sequelae) [24]. Finally, ADRs related to the main cardiac PTs used for reporting CAP cardiotoxicity in EV were analyzed. 

#### 2.3.2. Disproportionality Analysis

To evaluate disproportionate reporting, the Reporting Odds Ratio (ROR) and 95% confidence interval (95% CI) could be calculated by comparison with other drugs used in common therapeutic areas and in similar clinical contexts, based on the EMA recommendation. Thus, a signal of disproportionate reporting is defined if the lower bound of the 95% confidence interval (CI) is greater than 1 and the number of ICSRs is greater than or equal to 5 [29]. The calculation was conducted with MedCalc Software Ltd (MedCalc Software Ltd, Ostend, Belgium). on https://www.medcalc.org/calc/odds_ratio.php (accessed on 10 August 2024) (Version 20.123) [30]. The ROR was estimated for the cardiotoxicity reported for different medical conditions with PTs, included in Table 1. For the analysis, other antitumor drugs were used as comparators (5-FU, BEV, IRI, OX, PAN) [31]. Moreover, because 5-FU is the active metabolite of CAP, the disproportionality analysis was performed for both drugs, to observe the possible differences between them. Initially, a signal assessment was conducted for reports under the SOC “Cardiac disorders” for CAP and 5-FU, using other antitumor drugs as comparators. Subsequently, disproportionality was evaluated for each cardiotoxic condition (myocardial infarction, arrhythmias, heart failure, and cardiomyopathy) associated with CAP and 5-FU, respectively. Reporting is considered disproportionate if the case count is ≥5 and the lower limit of the 95% confidence interval exceeds 1.0 [31]. All reports with CAP, 5-FU, BEV, IRI, OX, and PAN mentioned as suspect, interacting or concomitant were included in these analyses. 

### 2.4. Ethics

No personal information was contained in the ICSRs and these analyses do not refer to any identifiable person. Therefore, this study did not require ethics board approval [32].

## 3. Results

### 3.1. Descriptive Analysis

According to data submitted in EV until 28 July 2024, 37,983 cases were reported for CAP, similar to 5-FU (*n* = 36,683), but inferior to BEV (*n* = 57,757) and to OX (*n* = 56,460). Compared to other drugs used for reference, CAP had a higher number of reports (IRI—*n* = 17,728 and PAN—*n* = 6950). No significant differences regarding the proportions of each age category were registered between CAP and the other drugs. Thus, most reports were registered for CAP in the 18–64 years (44.8%) and 65–85 years (33.3%) groups (Table 2), similar to the other drugs. An interesting situation could be observed for CAP regarding the sex of patients. In the CAP group, the majority of ICSRs were submitted for females (56.8%), in comparison to 5-FU (43.2%), IRI (38.9%), OX (42.7%), and PAN (31.4%). The majority of ICSRs were reported from non-EU countries for CAP and for all other drugs used as references. HP was the most frequent category of reporters submitting ICSRs in EV for CAP and for the other antitumor drugs of interest. 

**Figure 1:**
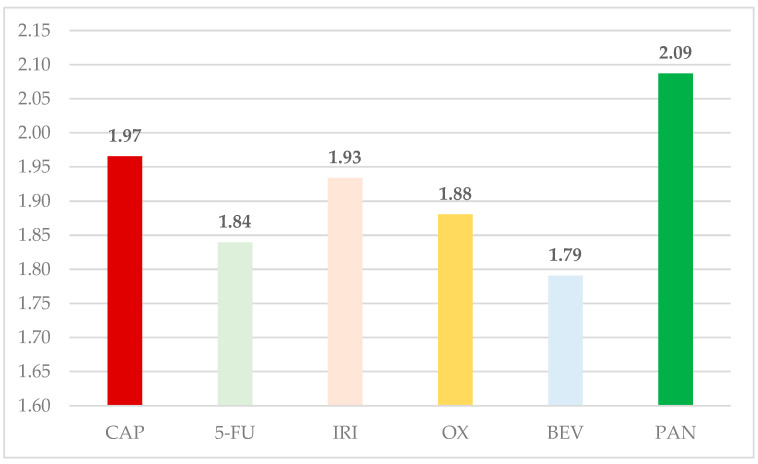
The comparative ratio of ADRs reported for each ICSR. 5-FU—5-fluorouracil; BEV—bevacizumab; CAP—capecitabine; IRI—irinotecan; OX—oxaliplatin; PAN—panitumumab.

The ratio between total ADRs and total ICSR submitted in EV was compared in Figure 1. For CAP (1.97), the ratio is higher than all other comparators, except for PAN (2.09). 

**Figure 2:**
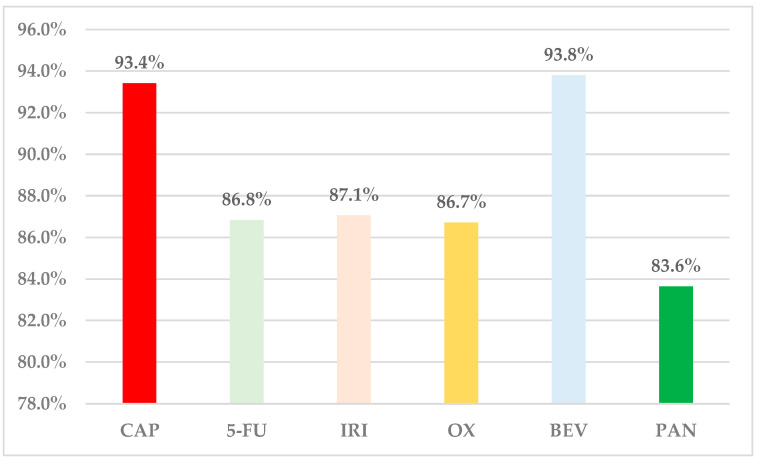
Structure of ADRs by seriousness. 5-FU—5-fluorouracil; BEV—bevacizumab; CAP—capecitabine; IRI—irinotecan; OX—oxaliplatin; PAN—panitumumab.

According to data submitted in EV, 93.4% of ADRs reported for CAP were serious: higher than PAN (83.6%), OX (86.7%), 5-FU (86.8%), and IRI (87.1%) (Figure 2).

Regarding the distribution of ADRs in SOC, higher proportions were observed for CAP in “Cardiac disorder” (3.4%) and “Gastrointestinal disorder" (15.0%) SOCs. A lower proportion was noticed for CAP regarding ADRs from “Infections and infestation” (3.8%) and “Nervous system disorder” (6.1%) SOCs (Table 3).

**Figure 3:**
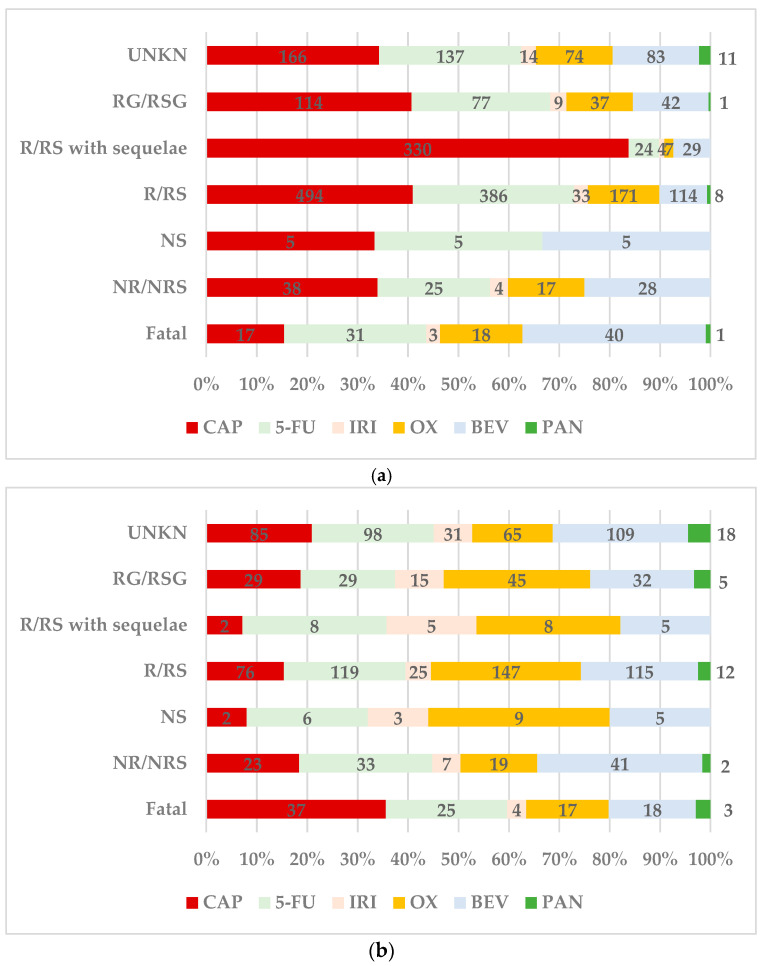

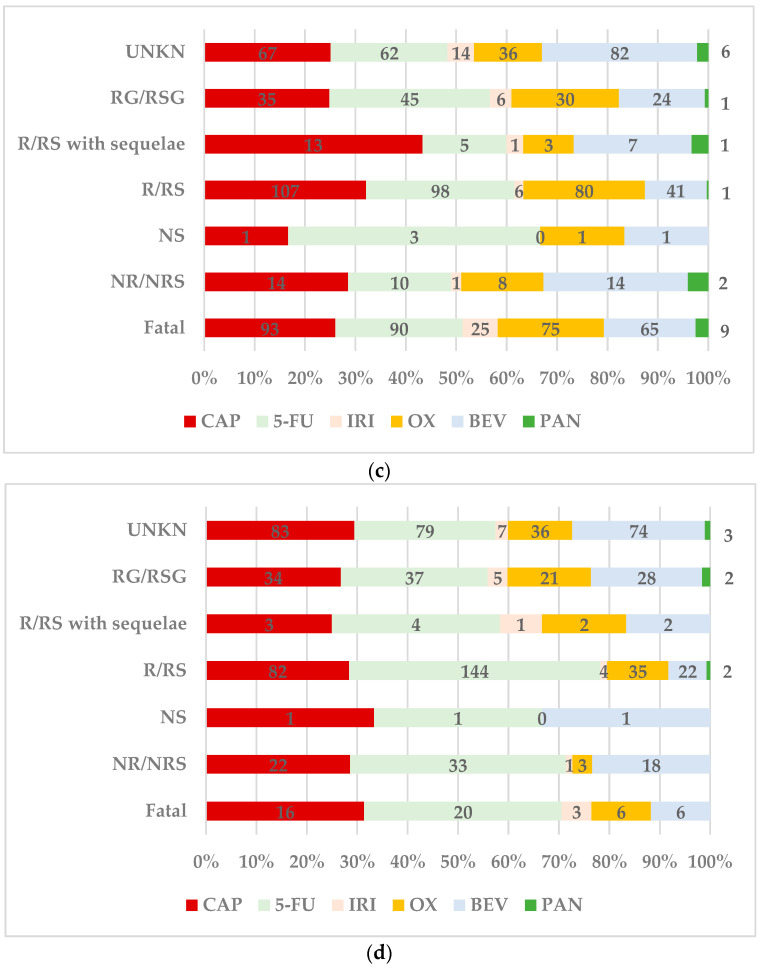
Distribution of ADRs by outcome. ADRs related to (**a**)—myocardial infarction; (**b**)—arrhythmias; (**c**)—heart failure; (**d**)—cardiomyopathy. 5-FU—5-fluorouracil; BEV—bevacizumab; CAP—capecitabine; IRI—irinotecan; OX—oxaliplatin; PAN—panitumumab; R—recovered; RS—resolved; NR—not recovered; NRS—not resolved; RG—recovering; RSG—resolving; UNKN—unknown.

70.4% of the total ADRs related to myocardial infarction reported after CAP use (*n* = 608) had a favourable outcome (R/RS or RG/RSG), a higher proportion than for the other drugs. Among the drugs of interest, the lowest percentage of ADRs leading to death was reported for CAP (*n* = 17, 2.0%) (Figure 3a). For arrhythmias produced by CAP, the highest proportion of ADRs with fatal outcomes (*n* = 37; 14.6%) compared to the other drugs was registered. A favourable outcome was reported for 41.3% of total ADRs (*n* = 105) related to arrhythmia produced by CAP (Figure 3b). The proportion of ADRs related to heart failure with fatal outcomes reported for CAP (*n* = 93, 28.2%) was similar to that for 5-FU (*n* = 90, 28.8%) and BEV (*n* = 65, 27.8%). The proportion of ADRs with favourable outcomes (*n* = 142, 43.0%) was also similar to 5-FU (*n* = 143, 45.7%) and OX (*n* = 110, 47.2%) and higher than the others (Figure 3c). Of the total ADRs related to myopathy reported for CAP, 6.6% (*n* = 16) had fatal outcomes, similar to 5-FU (*n* = 20, 6.3%) and OX (*n* = 6, 5.8%). The ratio of cases with favourable outcomes reported for CAP (*n* = 116, 48.1%) was lower than for 5-FU (*n* = 181, 56.9%), OX (*n* = 56, 54.4%), and PAN (*n* = 4, 57.1%) (Figure 3d).

**Figure 4:**
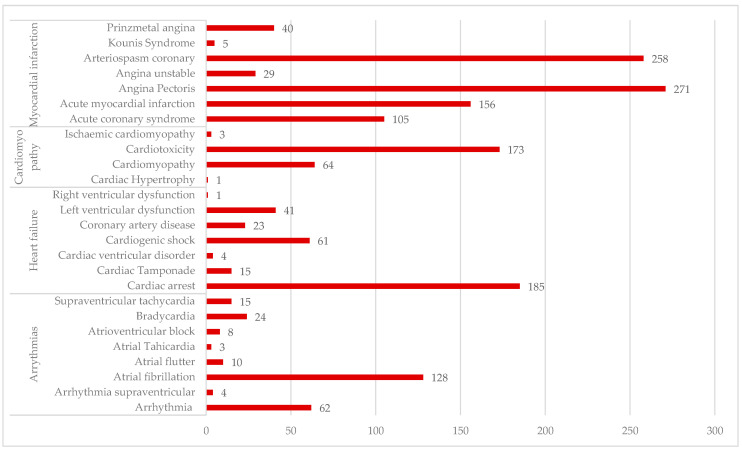
ADRs related to main cardiac PTs used for reporting in EV.

Figure 4 presents the distribution of the main cardiac PTs reported for CAP use. More than half of the total ADRs identified for the analysed medical conditions (*n* = 1689) are related to myocardial infarction (*n* = 864). ADRs for the other three medical conditions are approximately the same: cardiomyopathy—*n* = 241; heart failure—*n* = 330; arrhythmias—*n* = 254. According to this data, the distribution of the more frequent PTs used for each medical condition is as follows:

(i) MI: “Angina pectoris” (*n* = 271), “Arterio-spasm coronary” (*n* = 258), “Acute myocardial infarction” (*n* = 156), and “Acute coronary syndrome” (*n* = 105)
(ii) HF: “Cardiac arrest” (*n* = 185)
(iii) Cardiomyopathy: “Cardiotoxicity” (*n* = 173)
(iv) Arrhythmias: “Atrial fibrillation” (*n* = 128).

### 3.2. Disproportionality Analysis

#### 3.2.1. Analysis of Signals Reported in SOC “Cardiac Disorders”

**Figure 5:**
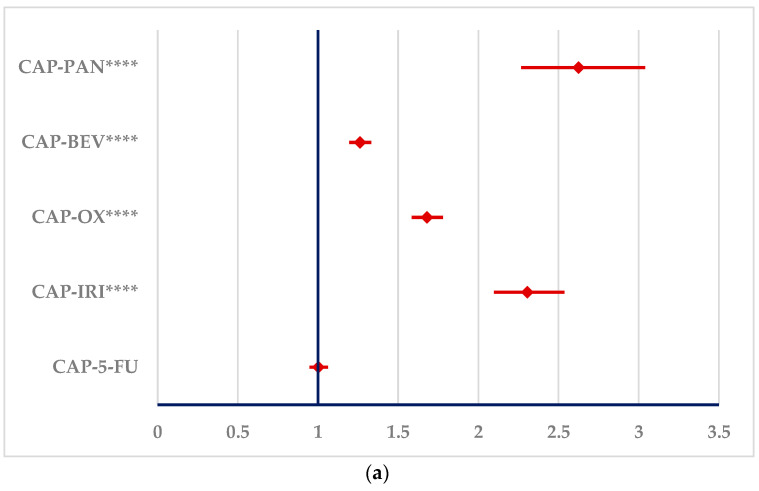

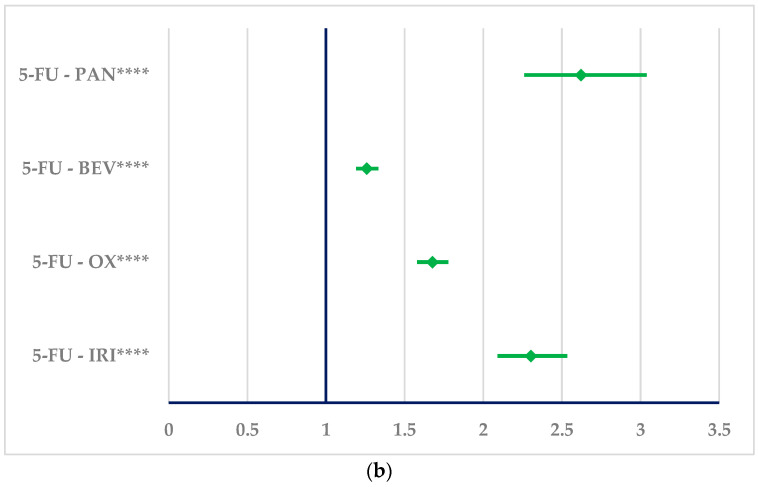
Disproportionality analysis of ADRs produced by CAP and 5-FU and reported in “Cardiac disorders” SOC. (**a**)—capecitabine; (**b**)—5-fluorouracil. 5-FU—5-fluorouracil; BEV—bevacizumab; CAP—capecitabine; IRI—irinotecan; OX—oxaliplatin; PAN—panitumumab. **** *p* ≤ 0.0001.

Figure 5 represents the disproportionality analysis of signals reported in SOC “Cardiac disorders”. Similar to 5-FU, CAP presents disproportionate signals compared to OX, IRI, BEV and PAN. On the other hand, a disproportionate signal could not be compared to 5-FU. 

#### 3.2.2. Analysis of Signals Related to Different Cardiac Diseases Reported After Capecitabine Use

**Figure 6:**
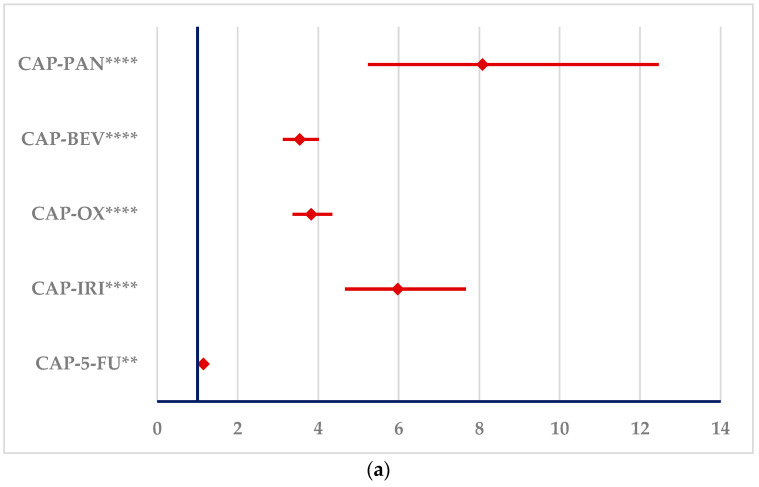

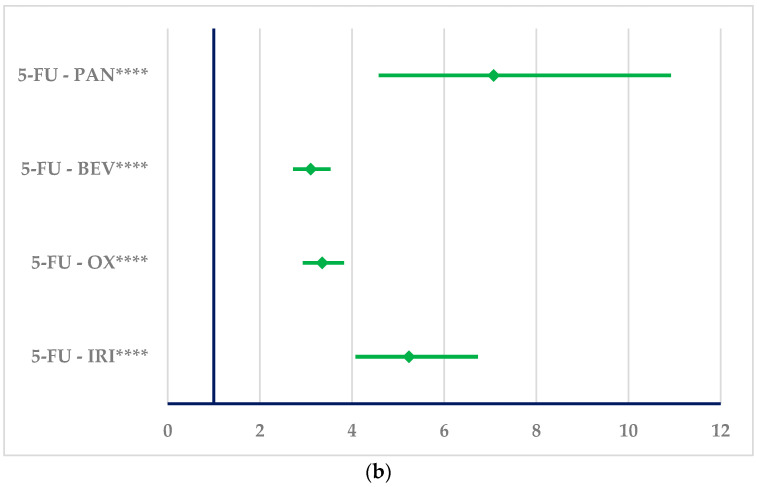
The signals for ADRs related to myocardial infarction produced by capecitabine and 5-fluorouracil. (**a**)—capecitabine (**b**)—5-fluorouracil. 5-FU—5-fluorouracil; BEV—bevacizumab; CAP—capecitabine; IRI—irinotecan; OX—oxaliplatin; PAN—panitumumab. ** *p* ≤ 0.01; **** *p* ≤ 0.0001.

According to Figure 6**,** CAP and 5-FU have a higher probability of reporting PTs related to myocardial infarction than IRI (ROR: 5.9799, 95% CI: 4.6623–7.6698), OX (ROR: 3.825 0, 95% CI: 3.3653–4.3474), BEV (ROR: 3.5402, 95% CI: 3.1220–4.0143), and PAN (ROR: 8.0770, 95% CI: 5.2372–12.4567). CAP also has a higher probability of reporting than 5-FU (ROR: 1.1418; 95% CI: 1.0323–1.2629).

**Figure 7:**
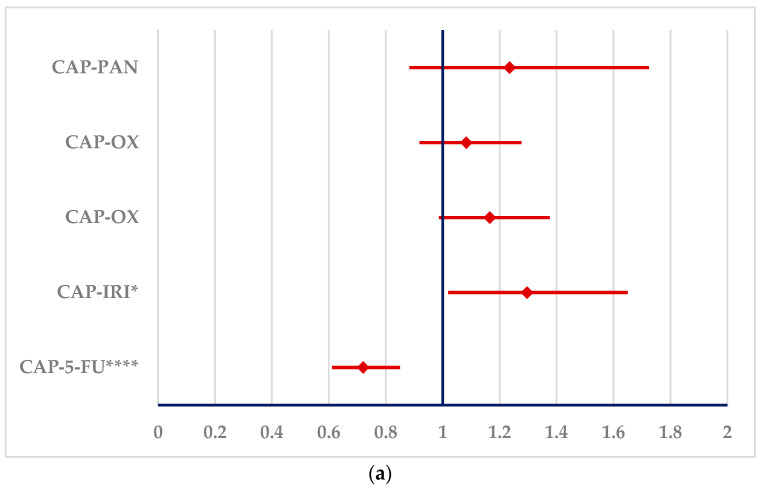

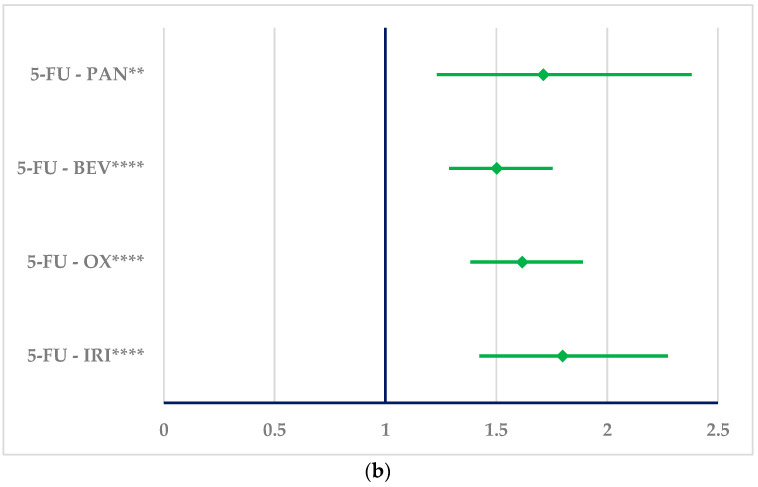
The signals in ADRs related to arrhythmias produced by capecitabine and 5-fluorouracil. (**a**)—capecitabine (**b**)—5-fluorouracil. 5-FU—5-fluorouracil; BEV—bevacizumab; CAP—capecitabine; IRI—irinotecan; OX—oxaliplatin; PAN—panitumumab. * *p* < 0.05; ** *p* ≤ 0.01; **** *p* ≤ 0.0001.

Arrhythmias produced by 5-FU could be reported with a higher probability than all other drugs. On the other hand, CAP has a higher probability of reporting arrhythmias only in comparison with IRI (ROR: 1.2971; 95% CI: 1.0196–1.6502). For CAP, a lower probability of reporting arrhythmias could also be observed than for 5-FU (ROR: 0.7211, 95% CI: 0.6112–0.8507) (Figure 7).

**Figure 8:**
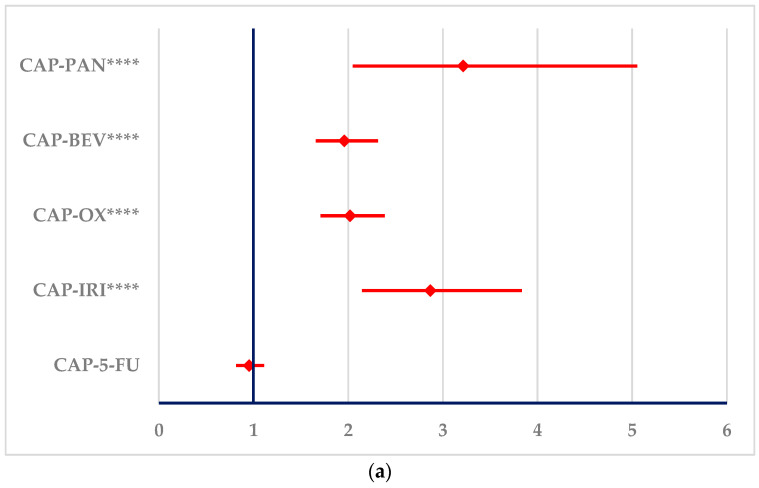

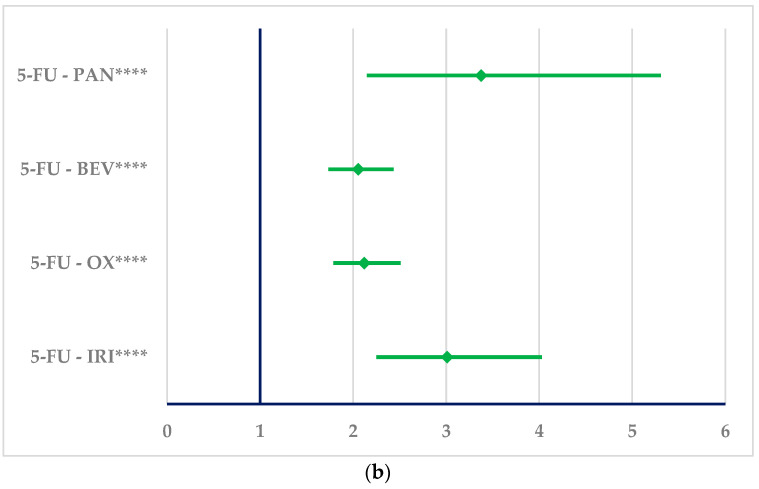
The signals in ADRs related to heart failure produced by capecitabine and 5-fluorouracil. (**a**)—capecitabine (**b**)—5-fluorouracil. 5-FU—5-fluorouracil; BEV—bevacizumab; CAP—capecitabine; IRI—irinotecan; OX—oxaliplatin; PAN—panitumumab. **** *p* ≤ 0.0001.

**Figure 9:**
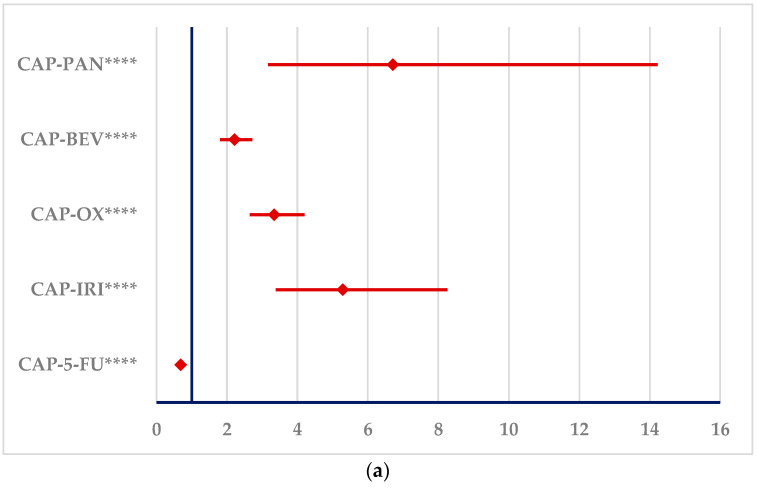

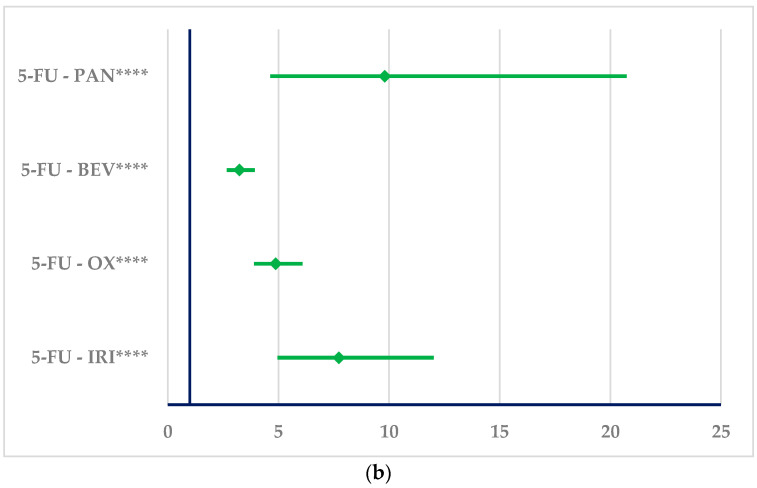
The signals in ADRs related to cardiomyopathy produced by capecitabine and 5-fluorouracil. (**a**)—capecitabine (**b**)—5-fluorouracil. 5-FU—5-fluorouracil; BEV—bevacizumab; CAP—capecitabine; IRI—irinotecan; OX—oxaliplatin; PAN—panitumumab. **** *p* ≤ 0.0001.

PTs related to heart failure (Figure 8) or cardiomyopathy (Figure 9) were reported with a higher probability for CAP and 5-FU than all other drugs. CAP presents a higher probability of reporting cardiomyopathy than 5-FU, but no differences between CAP and 5-FU could be observed for PTs related to heart failure.

## 4. Discussion

Our study highlights the distribution of cases reported in the EV database for CAP in comparison with other cytostatics for targeted therapies, such as 5-FU, OX, IRI, BEV, and PAN, respectively. The data analysed show a similar number of reported cases for CAP, as well as for 5-FU, but fewer reports are observed compared to OX and BEV. CAP has more reports than IRI and PAN.

More reports were identified in EV for OX and BEV than for CAP. There is a difference in reporting, possibly triggered by the diverse mechanisms of action and the different safety profiles of the drugs. OX is a platinum agent, having as its main side effect severe peripheral neurotoxicity, which can be cumulative, a reason why both patients and doctors report these adverse reactions more frequently [33]. BEV inhibits the vascular endothelial growth factor (VEGF), generating ADRs, such as arterial hypertension, thromboembolic events and gastrointestinal perforations, and negatively influencing the quality of life of patients [34,35].

In our study, more reports were registered for CAP compared to IRI and PAN, probably because of the frequent ADRs, but also because of the profiles of the well-known adverse reactions. CAP can present unpredictable and varied adverse reactions, especially cardiac and gastrointestinal toxicities, which are reported in large numbers on the EV platform [36]. PAN is a monoclonal antibody that acts against the epidermal growth factor receptor (EGFR) [37], which presents, especially, dermatological adverse reactions, such as less severe and frequent skin rashes, compared to cytostatic treatments. Its use is limited to stage IV disease, which may explain the small number of cases reported in EV [38,39].

It is important to mention that the distribution of cases reported in EV may vary depending on the differences in the clinical use of these drugs. Both CAP and 5-FU are used as a large proportion of treatments against colon cancer, while OX and BEV are treatments that are considered useful, especially in combination with other chemotherapeutics, which explains the important number of reports [37]. In advanced stages, PAN, BEV, and IRI are mainly used, thus reducing the probability of reporting frequent ADRs [40]. 

According to the data analysis, the highest number of reported ADRs occurs in patients aged 18–64 years with proportion of 44.8%, and 65–85 years at 33.3%, probably because there is a more frequent use of oncological treatments in this age group [41]. In the case of patients between the ages of 18 and 64, the detection and treatment of cancers are relatively fast, and therapies are more aggressive due to the patients’ better health and their ability to tolerate intensive chemotherapy. Among patients in the 65–85 age group, the use of oncological treatments is higher due to the incidence of neoplasia that increases with age [42,43]. It is a well-known fact that the tolerance of elderly patients to oncological treatments is reduced due to associated pathologies on the one hand and to the decrease in the physiological metabolism of drugs on the other [44,45,46]. 

Further, it was observed that the adverse effects induced by CAP are more frequent in women than in men, in contrast to the toxicity profiles observed for 5-FU, IRI, OX, and PAN [47,48]. A study by Milano et al. demonstrates that the hepatic clearance for 5-FU is lower in women than in men, with increased accumulation and exposure of the drug in the body [49]. In comparison to men, for women the ratio of adipose tissue to muscular tissue is higher, and the body fluid volume is lower, which demonstrates the impairment of the distribution volume of the drug [50,51]. From the point of view of the enzymes that metabolize CAP, such as dihydropyrimidine dehydrogenase (DPD), it has been observed that a partial deficiency may appear in women, which increases the digestive toxicity of this drug [52]. The study by Schünemann et al. demonstrates that women report ADRs more frequently than men, who tend to neglect certain symptoms [41]. 

According to EV data, the largest share of ADR reporters is represented by medical professionals. This is explained by the necessity that oncological treatments are to be administered in hospitals, or under very careful monitoring by specialized personnel if the patients follow a drug treatment at home. Thus, the medical staff are directly involved in reporting ADRs. Moreover, in some countries, there are very good procedures established, which require that the medical personnel report adverse effects [22,53,54]. 

The study highlights more frequent ADR reporting outside the European Economic Area (EEA) compared to EEA regions. This can be explained by factors related to drug consumption, reporting protocols, as well as the large volume of patients treated in regions outside the EEA [55,56]. The therapeutic protocols are similar globally, yet their application varies between the EEA and non-EEA regions, and certain drugs can be used extensively, thus increasing the incidence of adverse reactions [57]. 

According to the EV database, serious ADRs were reported in 93.4% of total ADRs for CAP, and this proportion is visibly higher compared to PAN (83.6%), OX (86.7%), 5-FU (86.8%) and IRI (87.1%). Only BEV outranks CAP in this regard. Hoff et al., demonstrated that patients who were administered oral pharmaceutical forms containing CAP had a higher incidence of ADRs compared to patients treated with intravenous 5-FU [58].

Polak et al. highlighted the fact that cardiac ischemia is associated with exposure to fluoropyrimidines, and the pathophysiological mechanism is demonstrated by the reduction of coronary blood flow due to vasospasm [59]. Gamelin et al. (2004) demonstrated the connection between the administration of fluoropyrimidines and the increased risk of cardiac arrhythmias, requiring careful monitoring of patients during treatment [60].

According to the present study, MI was more frequent in patients who followed treatment with CAP, compared to the drugs analysed in the studies. The data suggest that fatal MI induced by treatment with CAP was significantly less frequent, a fact that is considered to be effective in the management of this pathology, reducing mortality [61]. In the study conducted by Johannes et al., patients were included in the treatment based on CAP. Of the total patients, 5.9% presented cardiotoxicity related to CAP. The combined treatment of CAP with OX and BEV also led to the highest risk of cardiotoxicity [62].

HF is one of the most severe side effects following treatment with CAP. The data suggest that more than a quarter of the ADRs had a fatal outcome, a worrying result that marks the severity of HF associated with CAP [63]. The proportion of adverse events with a favourable outcome in patients treated with 5-FU and OX is higher compared to other treatments, which indicates that HF management is properly managed [44].

Another study highlights a case of CAP—induced cardiogenic shock. Thus, Andrew’s study presents the case of a patient diagnosed with appendicular mucinos adenocarcinoma, who developed cardiogenic shock 5 days after the start of capecitabine administration, being successfully treated by supportive care with dopamine, milrinone, noradrenaline and levosimendan [64].

The reported myopathies associated with treatment with CAP may be determined by cellular toxicity, impairment of cellular metabolism and the production of toxic metabolites. These events are rare and transitory [58]. 

Maharsy et al. indicate that MI caused/induced by fluoropyrimidines is associated with coronary vasospasm, the main triggering mechanism of acute myocardial ischemia [44].

Polka et al. demonstrated in 2022 that 4–10% of patients develop cardiotoxicity during treatment with fluoropyrimidines. In cases like this, the rate of MI is higher in comparison to cardiac pathologies [65].

Angina pectoris (*n* = 271), coronary arterio-spasm (*n* = 258) and cardiac arrest (*n* = 185), are the most frequent ADRs reported, these being important markers of cardiotoxicity associated with fluoropyrimidine-based chemotherapy. Moreover, they can have unpredictable developments regarding patients’ safety [23]. A prospective study that followed cardiotoxicity during exposure to 5-FU by Holter monitoring reported that 14% of subjects showed ischemic changes on Holter, 5.6% acute coronary syndromes and 1.8% asymptomatic arrhythmias; in addition, 75% of the ischemic changes were asymptomatic [66]. 

Kounis syndrome was reported in five cases in the EV database. Kazuhiko Kido at al. describe the case of a patient who developed Kounis syndrome approximately 5 months after the start of CAP treatment. The diagnosis was based on high levels of histamine, interleukin (IL)—6 and IL—10 [67]. 

The analysis of disproportionality marks the increased probability of reporting cardiac disorders for CAP and 5-FU, a common characteristic of fluoropyrimidines [68]. They have been well documented in specialized literature and it has been shown that these drugs can induce myocardial ischemia, angina pectoris, myocardial infarction, heart failure, arrhythmias and cardiomyopathies, whose causal mechanism is coronary vasospasm, myocardial ischemia, oxidative stress, etc. [39,69]. Our results are consistent with the results published by Xin Chen et al. highlighting that 80% of cancer survivors face chronic pathologies associated with oncological treatments during their lifetime. Specifically, cardiovascular diseases occupy the second place in terms of morbidity and mortality after exposure of patients to oncological treatments, such as: fluoropyrimidines, anthracyclines, kinase inhibitors, proteasome inhibitors, etc. [70]

Pre-clinical studies have identified the active (5-FU) and inactive metabolites of CAP, confirming its activity on xenograft and animal models, and the therapeutic dose was established [71,72]. 

Pre-marketing studies have some limitations regarding the short-term study duration and the limited number of patients included, even though these studies are rigorously conducted. Generally, patients are selected based on different characteristics, but their group is not fully representative of the entire population. In this context, continuously performed post-marketing evaluations based on real-world data add to the pre-clinical and clinical evidence and improve safety assessment by detecting some rare or long-term ADRs [73,74,75]

In this context, it is essential to monitor the cardiac function during fluoropyrimidine treatment. An example in this regard can be found in the article published by McAndrew et al., in which two cases of cardiogenic shock are reported shortly after the administration of CAP. The cases in point highlight the importance of rapid and accurate diagnosis, immediate discontinuation of CAP and multidisciplinary collaboration with specialists of cardio-oncology, these being essential measures in saving the patients [76]. Also, patients with cardiovascular risk factors or pre-existing cardiovascular pathologies must be evaluated and monitored by ECG and cardiac ultrasound to prevent fatal events [39,77]. In support of patients, cardio-oncology has been rapidly developed in recent years, being indispensable in the personalized and multidisciplinary treatment of patients undergoing potentially cardiotoxic treatments [78].

Finding effective antineoplastic molecules with moderate side effects is a general desire of the scientific community. New strategies used in colorectal cancer include monoclonal antibodies (e.g., trastuzumab) and kinase inhibitors (e.g., tucatinib). In 2023, the FDA approved the combination of trastuzumab and tucatinib for HER2-positive, chemotherapy-refractory, RAS wild-type unresectable, or metastatic colorectal cancer [75]. 

The *Tucatinib Plus Trastuzumab in Patients With HER2+ Colorectal Cancer* clinical study (NCT03043313) shows a lower incidence of cardiotoxicity for tucatinib and trastuzumab. A single case of unstable angina in 86 patients (1.16%) included in Cohort A (Tucatinib + Trastuzumab) and one case of heart failure in 28 patients (3.57%) included in Cohort C (Tucatinib Monotherapy) were reported [79]. However, trastuzumab presents cardiotoxicity by reducing the left ventricular ejection fraction [80]. Our study could be useful in investigating the cardiotoxicity of trastuzumab in combination with tucatinib and CAP in colorectal cancer.

### Limitations of the Study

Data collected from large pharmacovigilance databases that record spontaneous reports has intrinsic limitations. First of all, the lack of some types of information essential in such studies (e.g., the number of patients administered a certain drug), does not allow an exact calculation of the incidence rate or a real estimate of the risk associated with the use of a drug. The monitoring of the safety profile is carried out by the permanent amassing of ADRs, so that underreporting, for various reasons, is another limitation that can affect the validity and accuracy of the results. The detection and management of safety signals, the evaluation of updated periodic safety reports, and the evaluation of post-authorization safety studies are essential in this process, and underreporting remains a problem for which solutions are being sought. Another limitation refers to the accuracy of the information obtained from the reports, which may be incomplete, inaccurate and lack essential details, or may be subject to errors as a result of data entry, because reporting can be carried out both by healthcare professionals and by patients or consumers. On the other hand, aggregated data accessed for the present study did not allow the construction of a predictive relationship between variables and the probability of an adverse reaction. Thus, an advanced statistical method for evaluating the signals could not be applied. Moreover, the lack of information from the reports regarding the patients’ medical history, pre-existing risk factors for cardiovascular diseases, the concomitant use of other drugs, and the criteria and methodology used to diagnose adverse reactions is also a limitation. 

## 5. Conclusions

Cardiac toxicity associated with CAP treatment is a subject of high interest in the field of oncology. The analysis of the EudraVigilance database has shown that treatment with fluoropyrimidines induces adverse cardiovascular reactions, especially myocardial infarction, heart failure and cardiomyopathies. Arrhythmias have been shown to be more frequent side effects associated with 5-FU when compared with CAP.

Cardiac monitoring remains the essential assessment for patients treated with these drugs, especially in the case of patients with cardiovascular comorbidities. To manage cardiotoxicity, especially when presenting acute chest pain, a detailed patient history is essential, covering risk factors and specific information on drug administration (dose, administration route, and date of the last treatment cycle). Key investigations include ECG to detect ischemic changes or arrhythmias, along with cardiac ultrasound, troponin levels, BNP measurement, and, if necessary, coronary angiography for comprehensive diagnosis. Adjustment of treatment dosages or its interruption may be necessary to prevent a major adverse effect.

## Figures and Tables

**Table 1:** PTs used for evaluation of cardiotoxicity.

Medical Condition	PT
Arrythmias	Arrhythmia
	Arrhythmia supraventricular
	Atrial fibrillation
	Atrial flutter
	Atrial tachycardia
	Atrioventricular block
	Bradycardia
	Supraventricular tachycardia
Heart failure	Cardiac arrest
	Cardiac Tamponade
	Cardiac ventricular disorder
	Cardiogenic shock
	Coronary artery disease
	Left ventricular dysfunction
	Right ventricular dysfunction
Cardiomyopathy	Cardiac Hypertrophy
	Cardiomyopathy
	Cardiotoxicity
	Ischaemic cardiomyopathy
Myocardial infarction	Acute coronary syndrome
	Acute myocardial infarction
	Angina pectoris
	Angina unstable
	Arterio-spasm coronary
	Kounis syndrome
	Prinzmetal angina

**Table 2:** Characteristics of records associated with capecitabine in EudraVigilance. EEA—European Economic Area; NS—not specified.

Characteristics	*N*	%
Age category		
NS	7837	20.6%
0–1 Month	12	0.0%
2 Months–2 Years	20	0.1%
3–11 Years	8	0.0%
12–17 Years	19	0.1%
18–64 Years	17,015	44.8%
65–85 Years	12,654	33.3%
More than 85 Years	418	1.1%
Sex		
Female	21,557	56.8%
Male	14,706	38.7%
NS	1.72	4.5%
Origin		
EEA	12,405	32.7%
NON-EEA	25,578	67.3%
NS	0	0
Reporter category		
Reporter		
HP	33,602	88.5%
Non-HP	4333	11.4%
NS	48	0.1%

**Table 3:** Distribution of ADRs by SOCs. 5-FU—5-fluorouracil; BEV—bevacizumab; CAP—capecitabine; IRI—irinotecan; OX—oxaliplatin; PAN—panitumumab.

SOC	CAP	5-FU	IRI	OX	BEV	PAN
Blood and lymphatic system disorders	11.7%	16.5%	16.2%	13.0%	7.5%	5.3%
Cardiac disorders	3.4%	3.4%	1.5%	2.1%	2.7%	1.3%
Congenital, familial and genetic disorders	0.3%	0.2%	0.2%	0.1%	0.1%	0.2%
Ear and labyrinth disorders	0.3%	0.3%	0.2%	0.2%	0.2%	0.1%
Endocrine disorders	0.2%	0.3%	0.1%	0.1%	0.5%	0.1%
Eye disorders	0.9%	0.9%	0.7%	1.0%	3.1%	1.8%
Gastrointestinal disorders	15.0%	13.7%	17.9%	11.5%	12.7%	8.9%
General disorders and administration site conditions	13.7%	12.7%	12.1%	11.3%	12.5%	11.9%
Hepatobiliary disorders	2.5%	2.0%	1.7%	2.1%	2.2%	1.6%
Immune system disorders	0.6%	0.9%	1.1%	5.5%	0.9%	1.1%
Infections and infestations	3.8%	4.5%	4.4%	2.2%	5.5%	7.9%
Injury, poisoning and procedural complications	3.6%	3.3%	3.5%	2.6%	5.8%	3.8%
Investigations	7.4%	7.0%	7.1%	8.0%	6.0%	4.1%
Metabolism and nutrition disorders	3.9%	3.7%	4.2%	2.5%	2.2%	5.7%
Musculoskeletal and connective tissue disorders	2.2%	1.4%	1.4%	1.8%	2.5%	1.1%
Neoplasms benign, malignant and unspecified (including cysts and polyps)	5.3%	4.4%	4.5%	2.0%	4.9%	7.1%
Nervous system disorders	6.1%	7.1%	6.6%	10.6%	7.4%	5.3%
Pregnancy, puerperium and perinatal conditions	0.0%	0.2%	0.0%	0.0%	0.0%	0.0%
Product issues	0.1%	0.2%	0.1%	0.1%	0.3%	0.0%
Psychiatric disorders	1.1%	0.9%	0.7%	0.7%	0.8%	0.5%
Renal and urinary disorders	1.9%	2.1%	2.0%	1.5%	4.0%	1.5%
Reproductive system and breast disorders	0.4%	0.3%	0.3%	0.2%	0.6%	0.3%
Respiratory, thoracic and mediastinal disorders	3.6%	4.9%	4.9%	8.1%	6.7%	5.9%
Skin and subcutaneous tissue disorders	9.0%	5.7%	5.2%	7.8%	3.4%	21.7%
Social circumstances	0.1%	0.1%	0.0%	0.1%	0.1%	0.1%
Surgical and medical procedures	0.3%	0.3%	0.2%	0.1%	0.3%	0.7%
Vascular disorders	2.5%	3.0%	3.1%	4.6%	6.9%	1.9%
Total	100.0%	100.0%	100.0%	100.0%	100.0%	100.0%

## Data Availability

Data are contained within the article.
